# Supplementary material for: Effect of malaria and HIV/AIDS co-infection on red blood cell indices and its relation with the CD4 level of patients on HAART in Bench Sheko Zone, Southwest Ethiopia
Source: PLoS One. 2022 Mar 4;17(3):e0263865. doi: 10.1371/journal.pone.0263865 (PMC8896715; doi:10.1371/journal.pone.0263865)
Supplement: S1 Appendix — (DOCX) [file pone.0263865.s001.docx]

# Appendix V:Questionnaire (English version)

**Code: ___________PART-I Questionnaires on Socio demographic Characters**

| **S.no**      **6.** | **Questions** | **Responses** |
| --- | --- | --- |
| 1. | Age of the study participant | _________Years |
| 2. | Sex | 1. Male 2. Female |
| 3. | Marital status**?** | 1. Married 2. Unmarried 3. Divorced 4. Widowed |
| 4. | Religion of the participants? | 1. Orthodox 2. Protestant 3. Muslim 4. Catholic 5. Others |
| 5. | Occupation of the participants? | 1. Governmental employee 2. Self employed 3. Merchant 4. Farmer 5. Other_____ |
| 6. | How much you earn? | ______ monthly  b. urban |
| **7.** | Place of residency? | 1. Rural 2. Urban |
| 8.  **9.** | Ethnic group of the participants  Suspected pregnancy? | 1. Bench 2. Sheko 3. Kaffa 4. Amhara 5. Other___ |
| 9. | Educational status of the participants? | 1. Uneducated 2. Primary 3. Secondary 4. Above secondary |
| 10. | Duration on ART? | 1. For 1 year 2. For 2 years 3. For 3 years 4. For 4 years 5. ≥5 years |
| 11. | ART Regimen you have been treated with? | 1. 1^st^Line ART drugs 2. 2^nd^Line ART drugs |

**PART-II Questions concerning about Knowledge of malaria transmission and prevention**

| 12 | Have you heard about malaria? | 1. Yes 2. No |
| --- | --- | --- |
| 13. | Is Malaria transmissible? | 1. Yes 2. No |
| 14. | What do you think about the means of malaria transmission? | 1. Mosquito bite 2. Body contact 3. Respiratory route 4. Other means |
| 15. | Is it possible to prevent Malaria? | 1. Yes 2. No |
| 16. | If your answer to question number 15 is yes what is/are the prevention methods?(you can choose more than just one method) | 1. Ant malarialTablets 2. Bed nets 3. Mosquito repellents 4. Environmental sanitation 5. Others |
| 17. | If your answer to question number 15 is bed nets use as a prevention method for malaria, how often do you use bed nets? | 1. Once in a week 2. Twice in a week 3. Three times in a week 4. ≥Four times in a week |

# Appendix VI: Questionnaire (በአማረኛ የተዘጋጄ ቃለመጠየቅ)

መለያ ( )ክፍል(አንድ)ማህበራዊና ሥነ ሕዝብ የሚመለከት ጥያቄ

1ኛ. ዕድሜ --------------ዓመት

2ኛ. ፆታ **1.** ወንድ 2. ሴት

3ኛ.የጋብቻሁኔታ 1.ያገባ 3 የፈታ/ች

2.ያላገባ 4. የሞተበት/ባት

4ኛ. ሃይማኖት 1. ኦርቶዶክስ 3.ሙስሊም

2. ፕሮተስታንት 4. ካቶሊክ 5. ሌሎች

5ኛ.የሥራ ሁኔታ 1.የመንግስት ሠራተኛ 2.በግል ተቀጣሪ 3.ነጋዴ 4.ገበሬ 5. ሌላ

6ኛ.የገቢ ሁኔታ --------------------ብር በወር

7ኛ. የሚኖሩበት አድራሻ 1.ገጠር 2. ከተማ

8ኛ.ብሔር 1. በንች 3. ከፋ

2. ሸኮ 4 አማራ 5 ሌላ

9ኛ. የትምርት ደረጃ 1.ያልተማረ 3. 2ኛ ደረጃ

2.የመጀመሪያ ደረጃ 4. ከ2ኛ ደረጃ በላይ

10ኛ. መድሃኒት ላይ የቆዩበት ዓመት 1. ለ1ዓመት 3 .ለ3ዓመት

2. ለ2 ዓመት 4. ለ4ዓመት 5.ለ5ዓመትና ከዚያም በላይ

11ኛ. መድሃኒት አይነት 1. የመጀመሪያ 2. ሁለተኛ

**ክፍል(ሁለት) ስለ ወባ በሽታ የሚመለከት ጥያቄ**

12ኛ. ስለ ወባ በሽታ ሰምተው ያውቃሉ? 1.አዎ 2.አይደለም

13ኛ. ስለወባ በሽታ መተላለፊያ መንገድ ያውቃሉ? 1.አዎ 2. አላውቅም

14ኛ. ለ13ኛ ጥያቄ መልስዎ አዎ ከሆነ የመተላለፊያ መንገዱ ምንድነው?

1. በትንኝ ንክሻ 2. የሰውነት ንክክ 3.በመተንፍሻ አካላት 4.በሌላ መንገድ

15ኛ. የወባ በሽታን መከላከል ይቻላል? 1. አዎ 2. አይቻልም

16ኛ. ለ15ኛ ጥያቄ መልስዎ አዎ ከሆነ የሚቻልበት መንገድ ምንድነው?

1. ክኒን በመጠቀም 3. የትንኝ ርጭትመጠቀም

2. አጎበር በመጠቀም 4. አካባቢን ማጽዳት 5. በሌላ መንገድ

17ኛ. ለ16ኛ ጥያቄ መልስዎ አጎበር መጠቀም ከሆነ በሳምንት ለምን ያህል ጊዜ ይጠቀማሉ?

1 አንድ ጊዜ 2 ሁለት ጊዜ 3 ሦስት ጊዜ 4 ከአራት ጊዜና ከዚያበላይ

**PART-III Questionnaires on clinical characteristics and Biochemical Parameters**

| 18. | Stage of HIV/AIDS? | 1. Stage I 2. Stage II 3. Stage III 4. Stage IV |
| --- | --- | --- |
| 19. | CD4 count in cells/ μl? | Recent CD4 _____ cells/μl |
| 20. | Species of malaria? | 1. P. falciparum 2. P. vivax 3. Mixed infection |
| 21. | Red blood cell count (RBC)? | __________ RBCX10^12/l^ |
| 22. | Hemoglobin (Hb)? | ____________ g/dl |
| 23. | Hematocrit (HCT)? | ____________% |
| 24. | Mean cell volume (MCV)? | ___________fl |
| 25. | Mean cell hemoglobin (MCH) | ____________Pg |
| 30. | Mean cell hemoglobin concentration (MCHC)? | _________g/dl |
| 31. | Red cell distribution width (RDW)? | ________SD% |
