# Supplementary material for: Effect of malaria and HIV/AIDS co-infection on red blood cell indices and its relation with the CD4 level of patients on HAART in Bench Sheko Zone, Southwest Ethiopia
Source: PLoS One. 2022 Mar 4;17(3):e0263865. doi: 10.1371/journal.pone.0263865 (PMC8896715; doi:10.1371/journal.pone.0263865)
Supplement: Attachment — Submitted filename: Response to reviewers.docx [file pone.0263865.s008.docx]

**Response to reviewers**

Dear reviewers, First and above all I highly thank you very much for your constructive comments that progresses me forward significantly from stage to stage. I believe that I learned a lot from every single comment that you provided. Furthermore I am ready to receive any comments regarding the manuscripts. Thanks for understanding me!!

Here under I attempt to address the previous and the current comments provided by you.

1. I minimized the abbreviations and in place where I am supposed to use them I defined them clearly before using them
2. I tried to minimize the inclusion bias among groups, particularly co-infected one; they were interviewed and assured that they were not sick of other chronic diseases. In this sense the co-infected group is in a similar state with the exception of acute malaria.
3. The study participants were duly acknowledged for their unreserved collaboration
4. Before the collection of both laboratory data and data from interview I secured the consent of the participants both in written and oral form( the detail is available in major document)
5. The detail sample size calculation procedure were followed
6. Yes the study have couple of limitations:-

6.1 It did not assessed cause and effect, because crossectional study design was employed 6.2 The CD4 count was not measured at the time of data collection, however secondary data was used from patients chart.

6.3 It would have been better if we had used PCR for diagnosis of malaria

7. The questionnaire tool (English version ) is attached at the last section of the document
8. The Ethical issue is secured from the Research Ethical Committee of Addis Ababa university and Bench Maji Health office
9. I assign a number for each line and I tried to correct spelling errors
10. Regarding the correlation of RBC indices and CD4 count the detail is located on table 4
11. All the spelling ,grammatical and abbreviations issue is secured
12. In material and method section I divided the subtitles with their respective heading
13. Tables in this manuscript is attached at final section of the document

**The correction of the current comment sent to me on Sep 9, 2021, 5:13 PM**

14. Based on the detail description of the study and its possible minimal side effects,
15. the patients get clarification and gave their signature on the provided space after reading
16. by themselves or hearing when data collector read to them
17. The overall procedure of securing informed consent is approved by Research and Ethics
18. Committee of Medical Physiology department, Addis Ababa University.
19. Detailed calculation of sample size was placed under method section under subtitle of
20. sample size determination (Page 5, from line 49-58)
21. The potential limitations of this study was:-
22. Cross-sectional study design by itself is a limitation because it doesn’t assess cause and
23. effect relationships as the longitudinal study design does.
24. Second, it would have been better if we
25. would have used PCR for malaria species identification.
26. The sample size was fair, although it was low relatively due to resource constraints.
27. However, it will be highly representative if the sample size would have been increased

Appendix V: Questionnaire (English version)

**Code: ___________PART-I Questionnaires on Socio demographic Characters**

| **S.no**          **6.** | **Questions** | **Responses** |
| --- | --- | --- |
| 1. | Age of the study participant | _________Years |
| 2. | Sex | 1. Male 2. Female |
| 3. | Marital status**?** | 1. Married 2. Unmarried 3. Divorced 4. Widowed |
| 4. | Religion of the participants? | 1. Orthodox 2. Protestant 3. Muslim 4. Catholic 5. Others |
| 5. | Occupation of the participants? | 1. Governmental employee 2. Self employed 3. Merchant 4. Farmer 5. Other_____ |
| 6. | How much you earn? | ______ monthly  b. urban |
| **7.** | Place of residency? | 1. Rural 2. Urban |
| 8.  **9.** | Ethnic group of the participants    Suspected pregnancy? | 1. Bench 2. Sheko 3. Kaffa 4. Amhara 5. Other___ |
| 9. | Educational status of the participants? | 1. Uneducated 2. Primary 3. Secondary 4. Above secondary |
| 10. | Duration on ART? | 1. For 1 year 2. For 2 years 3. For 3 years 4. For 4 years 5. ≥5 years |
| 11. | ART Regimen you have been treated with? | 1. 1^st^Line ART drugs 2. 2^nd^Line ART drugs 3. Other Type if there |

**PART-II Questions concerning about Knowledge of malaria transmission and prevention**

| 12 | Have you heard about malaria? | 1. Yes 2. No |
| --- | --- | --- |
| 13.   a. | Is Malaria transmissible? | 1. Yes 2. No |
| 14.   b. | What do you think about the means of malaria transmission? | 1. Mosquito bite 2. Body contact 3. Respiratory route 4. Other means |
| c.   15. | Is it possible to prevent Malaria? | 1. Yes 2. No |
| 16. | If your answer to question number 15 is yes what is/are the prevention methods?(you can choose more than just one method) | 1. Ant malarialTablets 2. Bed nets 3. Mosquito repellents 4. Environmental sanitation 5. Others |
| 17. | If your answer to question number 15 is bed nets use as a prevention method for malaria, how often do you use bed nets? | 1. Once in a week 2. Twice in a week 3. Three times in a week 4. ≥Four times in a week |

**PART-III Questionnaires on clinical characteristics and Biochemical Parameters**

| 18. | Stage of HIV/AIDS? | 1. Stage I 2. Stage II 3. Stage III 4. Stage IV |
| --- | --- | --- |
| 19. | CD4 count in cells/ μl? | Recent CD4 _____ cells/μl |
| 20. | Species of malaria? | 1. P. falciparum 2. P. vivax 3. Mixed infection |
| 21. | Red blood cell count (RBC)? | **__________** RBCX10^12/l^ |
| 22. | Hemoglobin (Hb)? | ____________ g/dl |
| 23. | Hematocrit (HCT)? | ____________% |
| 24. | Mean cell volume (MCV)? | ___________fl |
| 25. | Mean cell hemoglobin (MCH) | ____________Pg |
| 26. | Mean cell hemoglobin concentration (MCHC)? | _________g/dl |
| 27. | Red cell distribution width (RDW)? | ________SD% |

**Appendix VI: Questionnaire (በአማረኛ የተዘጋጄ ቃለ መጠየቅ)**

መለያ (	)ክፍል (አንድ) ማህበራዊና ሥነ ሕዝብ የሚመለከት ጥያቄ

1ኛ. ዕድሜ	--------------ዓመት

2ኛ. ፆታ	**1.** ወንድ	2. ሴት

3ኛ. የጋብቻ ሁኔታ	1. ያገባ	3 የፈታ/ች

2. ያላገባ	4. የሞተበት/ባት

4ኛ. ሃይማኖት	1. ኦርቶዶክስ	3. ሙስሊም

2. ፕሮተስታንት	4. ካቶሊክ	5. ሌሎች

5ኛ. የሥራ ሁኔታ	1. የመንግስት ሠራተኛ	2. በግል ተቀጣሪ 3. ነጋዴ	4. ገበሬ

6ኛ. የገቢ ሁኔታ	--------------------ብር በወር

7ኛ. የሚኖሩበት አድራሻ	1. ገጠር	2. ከተማ

8ኛ. ብሔር	1. በንች	3. ከፋ

2. ሸኮ	4 አማራ	5 ሌላ

9ኛ. የትምርት ደረጃ	1. ያልተማረ	3. 2ኛ ደረጃ

2. የመጀመሪያ ደረጃ	4. ከ2ኛ ደረጃ በላይ

10ኛ. መድሃኒት ላይ የቆዩበት ዓመት 1. ለ1ዓመት	3 .ለ3ዓመት

2. ለ2 ዓመት	4. ለ4ዓመት 5.ለ5ዓመትና ከዚያም በላይ

11ኛ. መድሃኒት አይነት	1. የመጀመሪያ	2. ሁለተኛ

**ክፍል( ሁለት) ስለ ወባ በሽታ የሚመለከት ጥያቄ**

12ኛ. ስለ ወባ በሽታ ሰምተው ያውቃሉ? 1. አዎ	2. አይደለም

13ኛ. ስለ ወባ በሽታ መተላለፊያ መንገድ ያውቃሉ? 1. አዎ	2. አላውቅም

14ኛ. ለ13ኛ ጥያቄ መልስዎ አዎ ከሆነ የመተላለፊያ መንገዱ ምንድ ነው?

1. በትንኝ ንክሻ	2. የሰውነት ንክክ	3. በመተንፍሻ አካላት	4. በሌላ መንገድ

15ኛ. የወባ በሽታን መከላከል ይቻላል? 1. አዎ	2. አይቻልም

16ኛ. ለ15ኛ ጥያቄ መልስዎ አዎ ከሆነ የሚቻልበት መንገድ ምንድ ነው?

1. ክኒን በመጠቀም	3. የትንኝ ርጭት መጠቀም

2. አጎበር በመጠቀም	4. አካባቢን ማጽዳት	5. በሌላ መንገድ

17ኛ. ለ16ኛ ጥያቄ መልስዎ አጎበር መጠቀም ከሆነ በሳምንት ለምን ያህል ጊዜ ይጠቀማሉ? 1 አንድ ጊዜ	2 ሁለት ጊዜ	3 ሦስት ጊዜ	4 ከአራ ጊዜና ከዚያ በላይ

**PART-III Questionnaires on clinical characteristics and Biochemical Parameters**

| 18. | Stage of HIV/AIDS? | 5. Stage I 6. Stage II 7. Stage III 8. Stage IV |
| --- | --- | --- |
| 19. | CD4 count in cells/ μl? | Recent CD4 _____ cells/μl |
| 20. | Species of malaria? | 4. P. falciparum 5. P. vivax 6. Mixed infection |
| 21. | Red blood cell count (RBC)? | __________ RBCX10^12/l^ |
| 22. | Hemoglobin (Hb)? | ____________ g/dl |
| 23. | Hematocrit (HCT)? | ____________% |
| 24. | Mean cell volume (MCV)? | ___________fl |
| 25. | Mean cell hemoglobin (MCH) | ____________Pg |
| 30. | Mean cell hemoglobin concentration (MCHC)? | _________g/dl |
| 31. | Red cell distribution width (RDW)? | ________SD% |
